# Late Development of Hagfish Vertebral Elements

**DOI:** 10.1002/jez.b.22489

**Published:** 2013-02-08

**Authors:** Kinya G Ota, Satoko Fujimoto, Yasuhiro Oisi, Shigeru Kuratani

**Affiliations:** 1 Laboratory of Aquatic Zoology, Marine Research Station, Institute of Cellular and Organismic Biology, Academia SinicaYilan, Taiwan; 2 Laboratory for Evolutionary Morphology, RIKEN Center for Developmental BiologyKobe, Japan; 3 Department of Biology, Graduate School of Science, Kobe UniversityKobe, Japan

## Abstract

It has been demonstrated recently that hagfishes, one of two groups of extant jawless vertebrates, have cartilaginous vertebral elements. Embryological and gene expression analyses have also shown that this group of animals develops a sclerotome, the potential primordium of the axial skeleton. However, it has not been shown unequivocally that the hagfish sclerotome truly differentiates into cartilage, because access to late-stage embryos and information about the cartilaginous extracellular matrix (ECM) are lacking for these animals. Here we investigated the expression patterns of the biglycan/decorin (*BGN*/*DCN*) gene in the inshore hagfish, *Eptatretus burgeri*. The homologue of this gene encodes the major noncollagenous component of the cartilaginous ECM among gnathostomes. We clearly identified the expression of this gene in adult vertebral tissues and in embryonic mesenchymal cells on the ventral aspect of the notochord. Taking into account that the sclerotome in the gnathostomes expresses *BGN/DCN* gene during the chondrogenesis, it is highly expected the hagfish *BGN/DCN*-positive mesenchymal cells are derived from the sclerotomes. We propose that hagfishes and gnathostomes share conserved developmental mechanisms not only in their somite differentiation, but also in chondrogenesis of their vertebral elements. *J. Exp. Zool. (Mol. Dev. Evol.) 320B:129–139, 2013*. © 2013 Wiley Periodicals, Inc.

Vertebral elements, namely cartilaginous or bony segmental nodules associated with the notochord, represent one of the crucial morphological characteristics that define the vertebrates (Janvier, 1996; Liem et al., 2001; Kardong, 2011). The status of this feature has been a central issue in the evolutionary origin of the vertebrates (Goodrich, 1930; Løvtrup, 1977; Forey and Janvier, 1993; Janvier, 1996). The hagfishes constitute one of two groups of extant jawless vertebrates (cyclostomes), which until recently had been believed to lack vertebral elements (Cole, 1905; Forey and Janvier, 1993; Liem et al., 2001; Kardong, 2011) (Fig. 1A). Although the monophyly of the cyclostomes is supported by molecular data from various sources (Stock and Whitt, 1992; Mallatt and Sullivan, 1998; Kuraku et al., 1999; Takezaki et al., 2003; Heimberg et al., 2010), the hagfishes are still placed basal to the other vertebrates in some textbooks, in which the absence of vertebral elements in the hagfishes is considered plesiomorphic, rather than the derived condition (Kardong, 2011; Liem et al., 2001).

**Figure 1:**
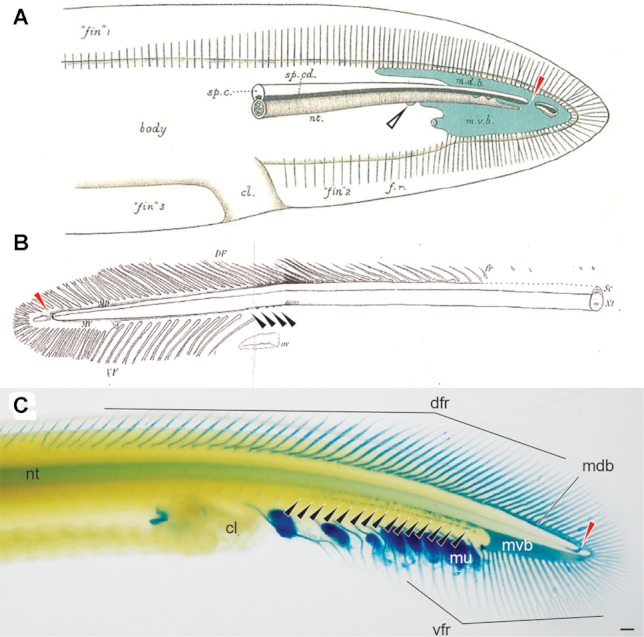
Caudal skeletons of three hagfish species. (**A**) Illustration of the caudal skeleton of *M. glutinosa* by Cole ('^05^). Median dorsal and ventral bars are indicated in blue. A single isolated cartilaginous nodule is identified (white arrowhead). (**B**) Illustration of the caudal skeleton of *Bdellostoma* species by Ayers and Jackson ('^00^). Cartilaginous nodules are identified on the ventral aspect of the notochord (black arrowheads). (**C**) Lateral view of the Alcian blue-stained caudal region of *E. burgeri*. At the postcloacal level, small cartilaginous nodules are located on the ventral side of the notochord (black arrowheads). In all these species, the median dorsal bar is attached to the notochord by the intermediate cartilaginous nodule (red arrowhead). Abbreviations: cl, cloaca; dfr, dorsal fin radials; mdb, median dorsal bar; mu, mucus gland; mvd, median ventral bar; nt, notochord; vfr, ventral fin radials. Scale bar, 1 mm.

According to the classical schema presented by Gadow (1895, 1933), the gnathostome vertebra consists of two dorsal and two ventral elements (see also Goodrich, 1930; Janvier, 1996). Of these, only the dorsal elements are present in the lamprey (Tretjakoff, 1926), but none of them were known in the hagfish species, although a few early researchers investigated their skeletal elements (Müller, 1834; Parker, 1883; Cole, 1905; Robson et al., 2000; Wright et al., 2001; see also Ota and Kuratani, 2010). An exceptional description was given by Ayers and Jackson (1900), who showed several anteroposteriorly arranged cartilaginous nodules on the ventral aspect of the caudal notochord of *Bdellostoma* more than a century ago (Fig. 1B). These cartilaginous structures had not been reexamined until our previous report (Ota et al., 2011).

In 2011, we reported that the Japanese inshore hagfish, *Eptatretus burgeri*, has vertebral elements (Ota et al., 2011) (Fig. 1C). These elements arise as small cartilaginous nodules on the ventral aspect of the notochord, reminiscent of the ventral elements of the gnathostomes (Gadow, 1933; Janvier, 1996). Our histological observations on embryos also suggested that the ventromedial part of the hagfish somite is transformed into mesenchyme that resembles the gnathostome sclerotome, the primordium of the vertebra (Christ et al., 2000; Ota et al., 2011). We also showed that the putative sclerotome in the hagfish expresses the *Twist* and *Pax1*/*9* genes encoding transcription factors involved in the differentiation of the sclerotome in the early and late pharyngular stages, respectively. These findings indicate that these cartilages are developmentally homologous with the vertebral elements of the gnathostomes. Thus, our observations suggest that the molecular mechanisms underlying early vertebral development are conserved between the hagfish and gnathostomes and that their evolutionary origins must date back 500 million years (Ota et al., 2011). However, some questions remain to be resolved: whether these putative sclerotomal cells truly form vertebral cartilage and whether their later developmental processes are also conserved between the gnathostomes and hagfishes. To answer these questions, it is necessary to study the late embryonic expression patterns of the genes encoding for the hagfish cartilaginous extracellular matrix (ECM).

The *Col2A1* genes encode the major representative protein components of the cartilaginous ECM. The genes have been thoroughly investigated and strong expression patterns in cartilaginous tissues and their primordia in the gnathostomes and lampreys have been reported (Zhang et al., 2006; see also Ota and Kuratani, 2009). However, none of the investigated hagfish *Col2A1* genes shows strong expression patterns in the cartilaginous tissues (Zhang and Cohn 2006; Ota and Kuratani, 2010), as observed in the lampreys and gnathostomes (Zhang et al., 2006; Ota and Kuratani, 2009). In addition, the noncollagenous cartilaginous ECM proteins are largely unknown in the hagfish. These data suggest that none of the known cartilaginous ECM protein-encoding genes are expressed at high levels in the hagfish; consequently, there are no available marker genes with which to investigate the processes of chondrogenesis in the hagfish vertebral elements.

Recent progress in our study of the developmental biology of the hagfishes has provided us with the sequence of the hagfish cartilaginous ECM gene. An embryonic cDNA library has been constructed, an expressed sequence tag (EST) project has been completed and the EST sequences are now freely available from a database (Takechi et al., 2011). Because this cDNA library was derived from a late-stage hagfish embryo, it is expected that candidate ECM genes that are expressed in the hagfish vertebral cartilaginous tissues will be found in this database. Moreover, we have successfully incubated a hagfish embryo for more than 200 days after egg deposition (Ota et al., 2011). This prolonged incubation has provided the prehatching stage of the hagfish embryo, which allowed us to investigate how the hagfish ECM genes are expressed during chondrogenesis in the embryonic vertebral tissues (Fig. 2).

**Figure 2:**
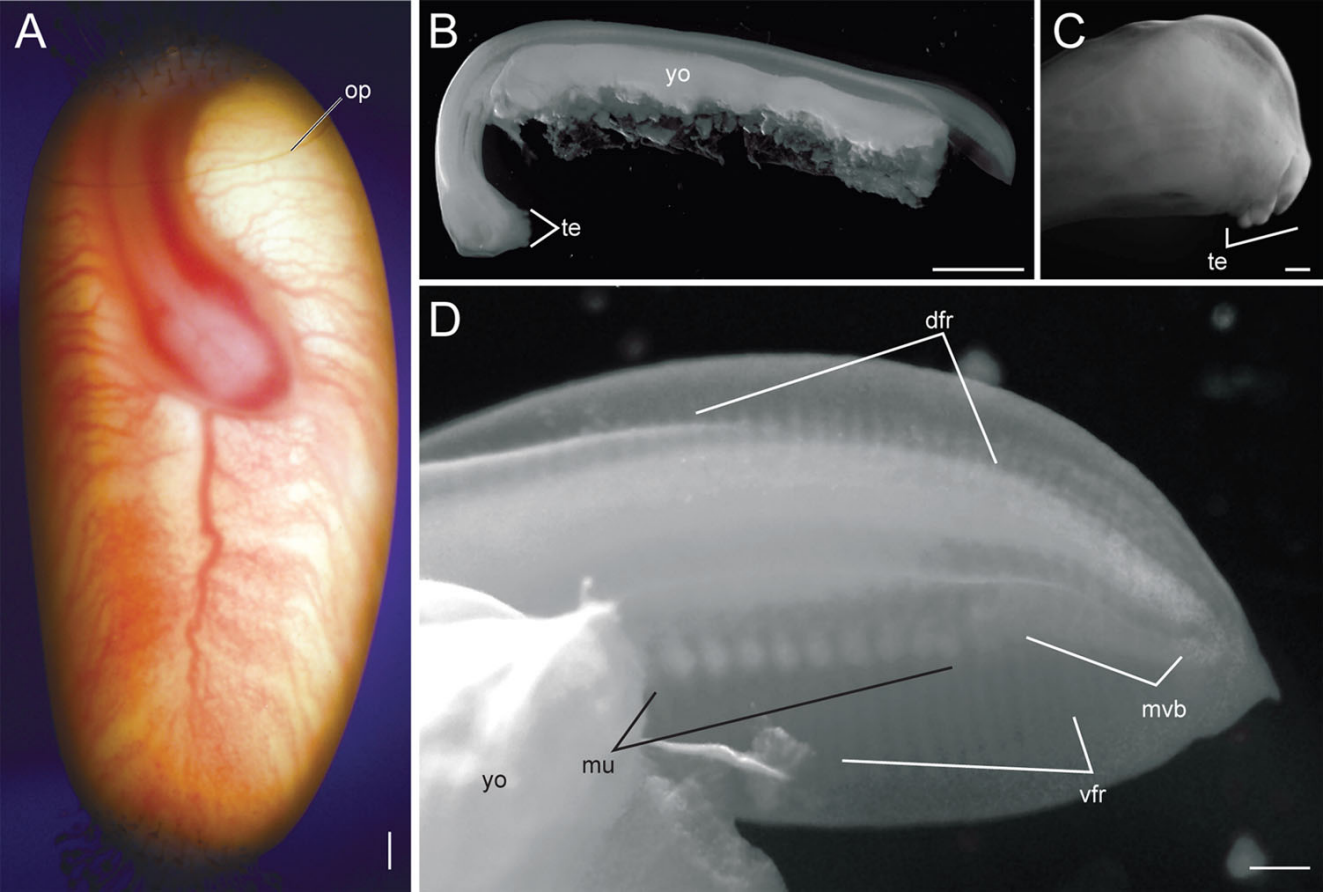
Prehatching stage of the *E. burgeri* embryo. (**A**) Ventral view of the 242-dpd embryo of the hagfish. The stage of this embryo is equivalent to that in Dean's figure 58 (Dean, 1899). (**B**) Lateral view of excised and cleared embryo. (**C**) Lateral view of the head (right side is anterior). (**D**) Higher magnification of the caudal area in (B). Abbreviations: dfr, dorsal fin radials; dpd, day postdeposition; mvb, median ventral bar; mu, mucus gland; op, opercular ring; te, tentacle; vfr, ventral fin radials; yo, yolk. Scale bars, 1 mm (A); 5 mm (B); 500 μm (C,D).

In this study, we screened the EST database and found a candidate gene encoding the major protein component of the hagfish cartilaginous ECM, biglycan/decorin (BGN/DCN). The *BGN* and *DCN* genes are known members of the class I small leucine-rich proteoglycan (SLRP) gene family and are expressed in the cartilaginous tissues of gnathostomes (Schaefer and Iozzo, 2008; Kalamajski and Oldberg, 2010). We analyzed the expression pattern of these genes in chondrocytes of the hagfish vertebral elements and in mesenchymal cells of prehatching-stage hagfish embryos. Based on these data, here we describe the early-to-late developmental processes of the hagfish vertebral elements and discuss their evolution.

## MATERIALS AND METHODS

### Sample Collection and Aquarium Maintenance

Adult male and female specimens of *E. burgeri* were collected using eel traps from a depth of 25–100 m in the Sea of Japan off Shimane and Yamaguchi Prefectures in September and October of 2008 and 2009. After the hagfish had been transferred to a laboratory aquarium (at 16°C) and were sexed by manipulation, around 100 adult specimens were maintained in a 1,000 L aquarium tank lined with some potentially favorable substrates including fine-grained sands and oyster shell, and kept at 16–17°C (see Ota and Kuratani, 2006, 2008; Ota et al., 2007, 2011). The deposited eggs were incubated individually in a plastic container (4 cm × 4 cm × 5 cm) in the same aquarium tanks (Ota et al., 2011). Fertilized eggs among those deposited were identified by visual inspection and prepared for further analysis.

### Survey of the EST Database and Identification of the *BGN*/*DCN* Gene

To retrieve the nucleotide and amino acid sequences of the cartilaginous ECM proteins from the hagfish embryonic ESTs (Takechi et al., 2011), BLAST searches were conducted against the Swiss-Prot and the National Center for Biotechnology Information (NCBI) nonredundant (nr) protein databases. From 161,482 ESTs, four sequences showed similarity to proteoglycan genes: three matching genes that encode epiglycan (Eb_eW_004_E04, Eb_eW_007_E12, and Eb_eW_008_D18) and one that encodes biglycan (Eb_eW_009_K05). The NCBI accession numbers for Eb_eW_004_E04, Eb_eW_007_E12, Eb_eW_008_D18, and Eb_eW_009_K05 are FY412279.1, FY413402.1, FY413755.1, and FY414270, respectively. To increase the number of informative sites used to identify the gene, the clone containing the Eb_eW_009_K05 sequence was resequenced between the 5′-untranslated region (UTR) and the 3′-UTR.

**Figure 3:**
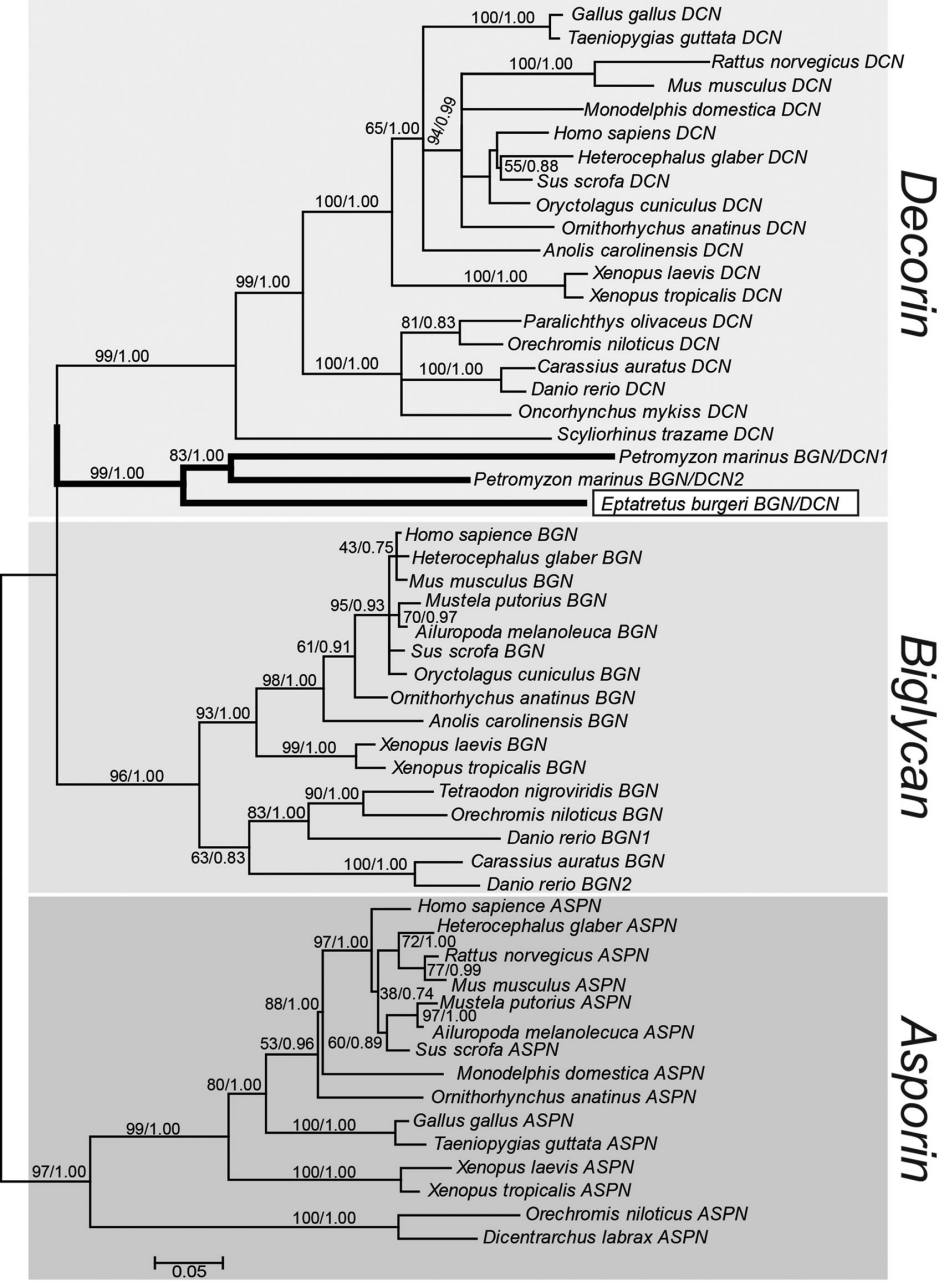
Phylogenetic tree of class I SLRP genes. Numbers at each node indicate the bootstrap (ML) and posterior probabilities (BI) (left and right numbers, respectively). The clades of the cyclostome class I SLRP genes are indicated with bold lines. The *EbBGN*/*DCN* gene is boxed. The vertical order of the sequences in the phylogenetic tree corresponds to that in the multiple-sequence alignment (Supplemental Fig. S1).

To identify those genes orthologous to the isolated fragments, sequence data involving class I SLRP genes, including those encoding biglycan (*BGN*), asporin (*ASPN*), and decorin (*DCN*), were obtained from the NCBI protein database using a BLAST search. A multiple-sequence alignment was generated using the CLUSTALW multiple alignment program (Supplementary Fig. S1; Thompson et al.,^94^). Preliminary neighbor-joining trees were constructed with MEGA (Kumar et al., 2008) and the sequences that showed extremely long branches on the preliminary neighbor-joining trees were removed from the multiple-sequence alignment to avoid long-branch attraction. To estimate the evolutionary relationships among the class I SLRP genes, a phylogenetic analysis was performed using two methods: the maximum likelihood (ML) and Bayesian inference (BI) methods. The alignment for the reconstruction of the trees contained 295 amino acid sites, and was derived from 23 vertebrate species (13 amniotes, two amphibians, six teleosts, one cartilaginous fish, one lamprey, and one hagfish). The *ASPN* genes were used as the outgroup, because these genes are known to have diverged from the other SLRP class I genes and because the amino acid sequences of the BGN and DCN proteins are closely related (Schaefer and Iozzo, 2008). The ML trees were reconstructed using 1,000 bootstrap replicates by applying the WAG model (Fig. 3). The BI analysis was based on two independent runs of 2 million generations, with samples taken from every 100 generations (Fig. 3); each run comprised one cold and three heated chains. The BI and ML analyses were performed using MrBayes and MEGA, respectively (Ronquist and Huelsenbeck, 2003; Kumar et al., 2008).

### Conventional Histological Analysis and In Situ Hybridization

Juvenile hagfish specimens (total length < 15 cm) and an embryo (see below) were anesthetized with MS-222, fixed by immersion in Serra's fixative, processed for paraffin wax sectioning using standard methods, cut into 6- to 8-μm-thick sections and stained with Alcian blue and hematoxylin and eosin (HE). The probes for in situ hybridization were prepared from PCR fragments amplified from the 5′ and 3′ ends of the isolated genes. These fragments were labeled using a digoxigenin (DIG) labeling kit (Roche Diagnostics K. K., Tokyo, Japan). In situ hybridization was performed in a Ventana automated machine (Roche Diagnostics K. K., Japan). A BlueMap NBT/BCIP substrate kit and ISH RED Counterstain (Roche Diagnostics K. K., Tokyo, Japan), a reagent equivalent to nuclear Fast Red, were used to detect the signal and for counterstaining, respectively. Several pilot in situ hybridizations were performed to select the appropriate DIG-labeled probes. Cartilaginous, muscular and other morphological elements are mainly reported using Cole's nomenclature (Ayers and Jackson, 1900; Cole, 1905; see also Brodal and Fänge, 1963; Jørgensen et al., 1998).

## RESULTS

### Prehatching Hagfish Embryos

Among the 42 fertilized eggs obtained in our previous work (Ota et al., 2011), one was incubated successfully for 242 days after its deposition (Fig. 2A). The blood vessels and embryonic structures were visible through the egg membrane (Fig. 2A). The head of this embryo was hanging over the yolk and the anterior quarter of the embryo could be seen from the ventral aspect (Fig. 2A). Many vitelline vessels were observed on the ventral side of the yolk (Fig. 2A). This embryo was close to the stage illustrated in Dean's Figure 58 (Dean, 1899).

Under the microscope, tentacles were observed around the mouth (Fig. 2B, C) and the mucus glands and segmental structure of the myoseptum were recognizable in the trunk region (Fig. 2D). Caudally, the dorsal and ventral cartilaginous fin radials and the median ventral bar could be observed with transmitted light (Fig. 2D). Although the vertebral elements could not be identified in our observation of the whole embryo—based on adult morphology—we expected that the cartilaginous nodules of the vertebral elements should develop on the ventral aspect of the notochord, along the anteroposterior axis between the median ventral bar and the yolk (Figs. 1C and 2D). It is also expected that chondrocytes or mesenchymal cells expressing cartilaginous ECM proteins can be detected in the same part of the caudal region of this embryo.

### Hagfish *BGN*/*DCN* Genes

Our survey of the hagfish EST database identified a clone (Eb_eW_009_K05) containing a class I SLRP gene. The insert of the clone comprised 1,176 base pairs of the coding region and the predicted amino acid sequence was 61.3% and 65.0% identical to two protein fragments encoded by SLRP genes of the lamprey (Supplementary Fig. S1); these two lamprey SLRP genes have been designated “biglycan-like genes 1 and 2” (Shintani et al., 2000). To determine the phylogenetic position of the hagfish class I SLRP gene, we constructed a molecular phylogenetic tree of class I SLRP genes, including *ASPN*, *BGN*, and *DCN* (Fig. 3).

Although the cyclostome SLRP genes showed affinity to the *DCN* genes of the gnathostomes (Fig. 3), we could not determine whether these cyclostome SLRP genes were orthologous to *BGN* or *DCN*. To improve the quality of the phylogenetic trees, we conducted molecular phylogenetic analyses with different datasets. However, these did not resolve the position of the clade containing the cyclostome SLRP genes. Therefore, we designated this newly isolated hagfish class I SLRP gene *EbBGN*/*DCN* and the SLRP genes of the lamprey *PmBGN*/*DCN1* and *PmBGN*/*DCN2*.

In all our phylogenetic analyses, *EbBGN*/*DCN* was located basal to the *PmBGN*/*DCN* clade with high supporting value (Fig. 3). *EbBGN*/*DCN* did not cluster with *PmBGN*/*DCN1* or *PmBGN*/*DCN2* on any phylogenetic tree. According to a previous report (Shintani et al., 2000), duplication of *BGN* occurred independently in the lamprey lineage after the divergence of *DCN* and *BGN*. Our phylogenetic analysis suggested the same scenario, with higher probability. Furthermore, although these three cyclostome *BGN*/*DCN* genes show relatively long branches, the supporting values at their nodes are relatively high (Fig. 3). Moreover, the clade of the cyclostome genes is isolated from the other *BGN* and *DCN* genes of the gnathostomes. This probably reflects the long period since the divergence of the hagfishes, lampreys and gnathostomes (Kuraku and Kuratani, 2006; Kuraku et al., 2009).

### Expression Patterns of the *EbBGN/DCN* Genes in the Hagfish Vertebral Elements

**Figure 4:**
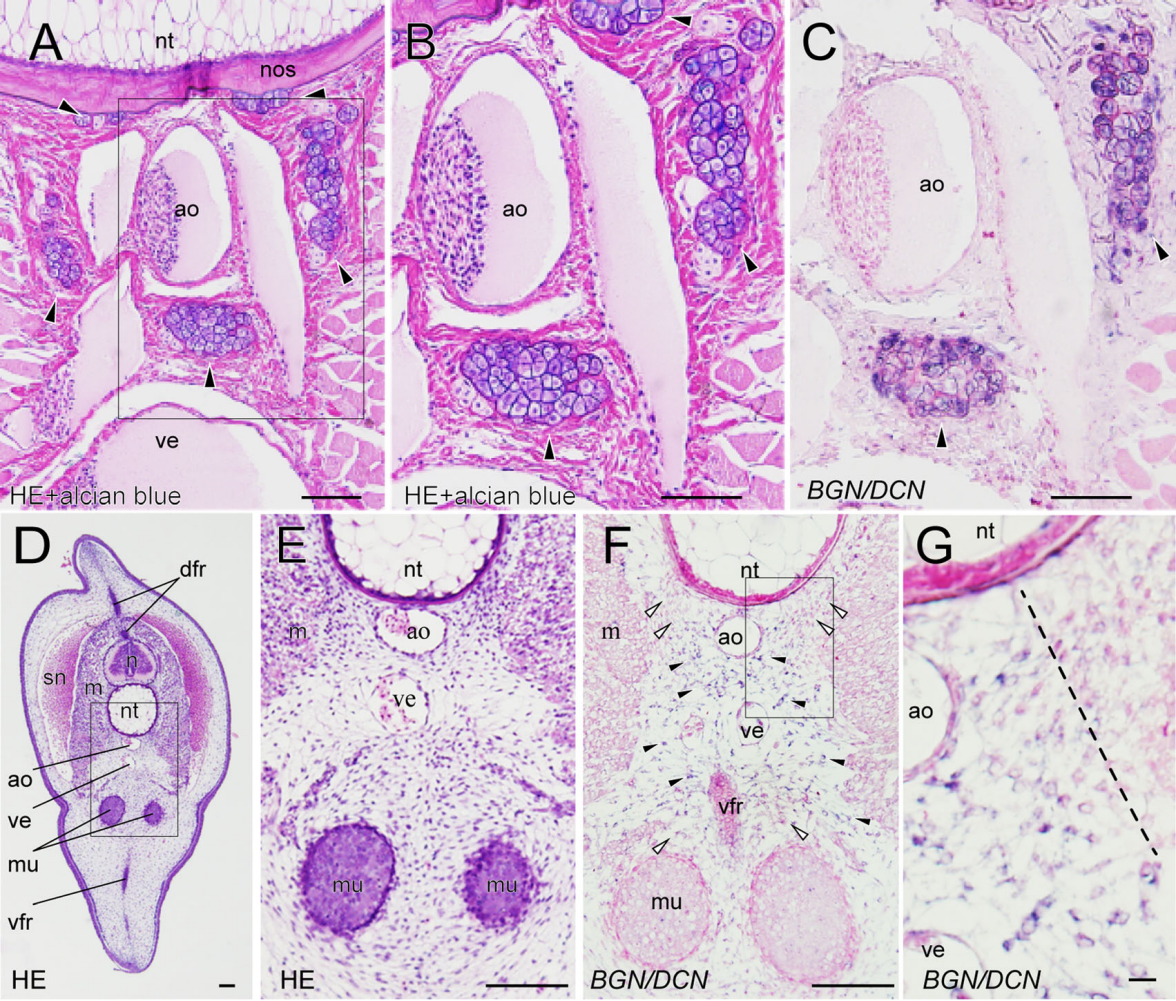
Histology and expression patterns of the *EbBGN*/*DCN* gene. (**A**–**C**) Transverse sections of an adult specimen of *E. burgeri* at the postcloacal level. (A) Cartilaginous nodules are located on the ventral aspect of the notochord and on the lateral and ventral aspects of the dorsal aorta (arrowheads). (B) Magnified view of the boxed area in (A). (C) Expression of *EbBGN*/*DCN* in the adult. Cartilaginous nodules strongly express the *EbBGN*/*DCN* gene. (**D**–**F**) Transverse section of the *E. burgeri* embryo at the postcloacal level. (D) Mesenchymal cells surround the dorsal and ventral aorta. (E) Magnified view of the boxed area in D. (F) Expression patterns of the *EbBGN*/*DCN* gene in the embryo. *EbBGN*/*DCN*-positive and -negative mesenchymal cells surrounding the dorsal aorta and posterior cardinal vein are indicated by black and white arrowheads, respectively. (**G**) Magnified view of the boxed area in (F). The dashed-line indicates the approximate boundary between *EbBGN/DCN*-positive and -negative areas. Abbreviations: ao, dorsal aorta; dfr, dorsal fin radials; m, myotome; mu, mucus gland; n, neural tube; nt, notochord; nos, notochordal sheath; sn, subcutaneous sinus; ve, posterior cardinal vein. Scale bars, 100 μm (A–F); 10 μm (G).

Although BGN and DCN are known to be the major protein components of the cartilaginous ECM in the gnathostomes (Schaefer and Iozzo, 2008; Kalamajski and Oldberg, 2010), it is still unclear whether the EbBGN/DCN proteins and their encoding mRNA are expressed in the hagfish vertebral cartilaginous tissues. Therefore, we investigated the expression patterns of the *EbBGN*/*DCN* gene in adult specimens (Fig. 4A–C).

First, we used conventional histology to analyze the postcloacal region of the adult specimens. In transverse views of HE- and Alcian blue-stained sections, five cartilaginous nodules were observed (Fig. 4A): one pair of elements attached to the ventral side of the notochord, another pair located on the lateral sides of the dorsal aorta and a single median element occupying a position between the dorsal aorta and posterior cardinal vein (Fig. 4A). These cartilaginous nodules were separated from each other by eosinophilic noncartilaginous connective tissues (Fig. 4B). At higher magnification, the chondrocytes in each nodule were seen to be surrounded by a thin Alcian blue-positive layer of ECM, indicating the presence of mucopolysaccharides (Fig. 4B). These results are consistent with anatomical observations of Alcian blue-stained whole-mount specimens of the adult hagfish (Fig. 1C).

We next conducted an in situ hybridization analysis of transverse sections at the same postcloacal level in adult specimens using an *EbBGN*/*DCN* riboprobe (Fig. 4C). We detected high levels of *EbBGN*/*DCN* transcripts inside the adult chondrocytes (Fig. 4C). Although subtle signals were also detected in the noncartilaginous connective tissues surrounding the cartilaginous nodules, the intensity of the signals clearly differed between the chondrocytes and these other connective tissues (Fig. 4C). Thus, the EbBGN/DCN protein is very probably one of the major ECM proteoglycans in the hagfish vertebral cartilage, providing a tool to investigate further the developmental processes of the hagfish vertebral elements in late embryos.

Histological sections of the prehatching-stage embryo were prepared to track back the development of the hagfish vertebral elements. Condensation of the mesenchymal cells of the dorsal and ventral fin radials was observed at the caudal level of the sections (Fig. 4D). Mesenchymal cells also occupied the ventral aspect of the notochord, surrounding the dorsal aorta and posterior cardinal vein (Fig. 4E). We also analyzed the expression pattern of the *EbBGN*/*DCN* gene at the same levels in prehatching embryos (Fig. 4F). The mesenchymal cells ventral to the notochord showed strong expression of the gene (Fig. 4F, G). The intensity of *EbBGN*/*DCN* expression in the mesenchyme around the dorsal aorta and posterior cardinal vein was also distinguishable from that in the other mesenchymal cells (Fig. 4G). Because the distribution of the *EbBGN*/*DCN* transcripts was consistent with that of the hagfish vertebral elements, these cells are very likely to differentiate into EbBGN/DCN-positive cartilage in the adult (Fig. 4C, F, G).

## DISCUSSION

**Figure 5:**
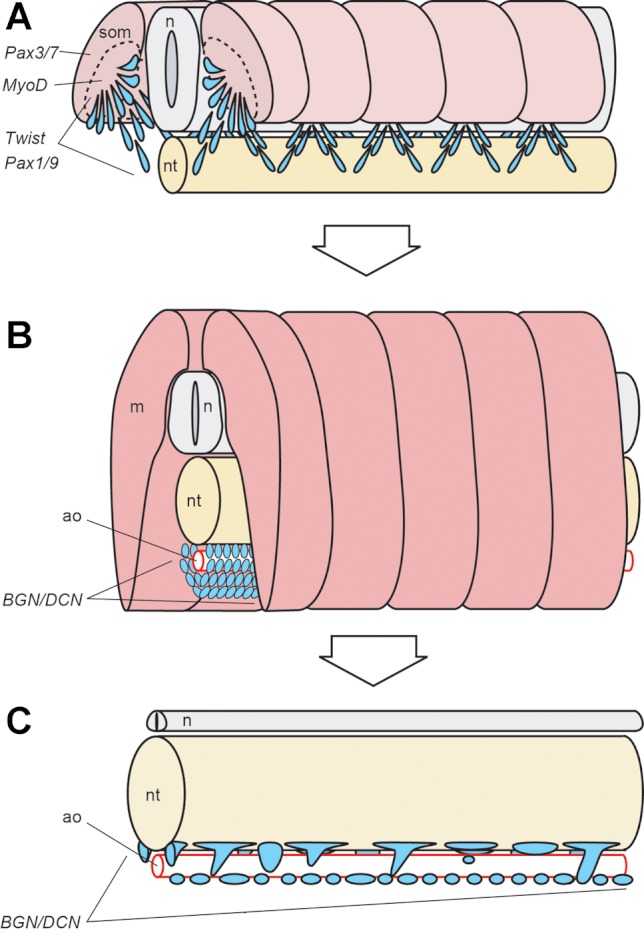
Scheme of the development of the vertebral elements of the hagfish. (**A**) Medial ventral somites differentiate into mesenchymal cells (blue) in the early pharyngular embryo. These mesenchymal cells express the *Twist* and *Pax1*/*9* genes. (B) A proportion of the mesenchymal cells migrate into the ventral aspect of the notochord and express the cartilaginous extracellular matrix genes, including *BGN*/*DCN*. (**C**) The mesenchymal cells differentiate into the cartilaginous nodules, continuously expressing the *BGN*/*DCN* gene. Abbreviations: ao, dorsal aorta; m, myotome; n, neural tube; nt, notochord; som, somite.

The results presented here strongly suggest that EbBGN/DCN is one of the major protein components of the ECM in the hagfish vertebrae, as in gnathostomes (Schaefer and Iozzo, 2008; Kalamajski and Oldberg, 2010). Our data also indicate that a number of embryonic mesenchymal cells on the ventral aspect of the notochord express the *EbBGN*/*DCN* gene strongly (Fig. 4F, G). In this study, we could not completely exclude the possibility that the hagfish vertebral elements are derived from other than sclerotomal cells. However, considering that the sclerotome in the gnathostomes produces a thick layer of cartilaginous ECM (Christ et al., 2000), it is reasonable to assume that the *EbBGN*/*DCN*-positive chondrocytes in the adult hagfish also derive from the *EbBGN*/*DCN*-positive mesenchyme in the late embryo (Fig. 4C, F, G). Based on this evidence, we will discuss the complete developmental processes of the hagfish vertebral elements (Fig. 5).

In previous studies, we have shown that the hagfish embryonic somite differentiates into three somitic derivatives: the dermomyotome, myotome, and sclerotome (Ota et al., 2007, 2011). These compartments are quite similar to those of the gnathostomes in their gene expression patterns (Christ et al., 2000; Buckingham and Vincent, 2009). In fact, strong expressions of *Pax3*/*7* on the dorsal side, *MyoD* on the medial side and *Pax1*/*9* and *Twist* on the ventromedial side of the somite have been detected (Ota et al., 2007, 2011). Considering the expression pattern of these genes, the developmental scenario of the hagfish vertebral elements can be summarized as follows. First, the ventral aspect of the somite is de-epithelialized and differentiates into the sclerotome, expressing *Pax1*/*9* and *Twist* and migrates to the ventral aspect of the notochord (Fig. 5A). The sclerotomal cells then surround the dorsal aorta and express *EbBGN*/*DCN* (Fig. 5B). Finally, these *EbBGN*/*DCN*-positive cells condense to form a cartilaginous nodule that maintains this pattern of gene expression (Fig. 5C). This scenario indicates that the initial processes of vertebral development are shared fundamentally by the hagfishes and gnathostomes.

**Figure 6:**
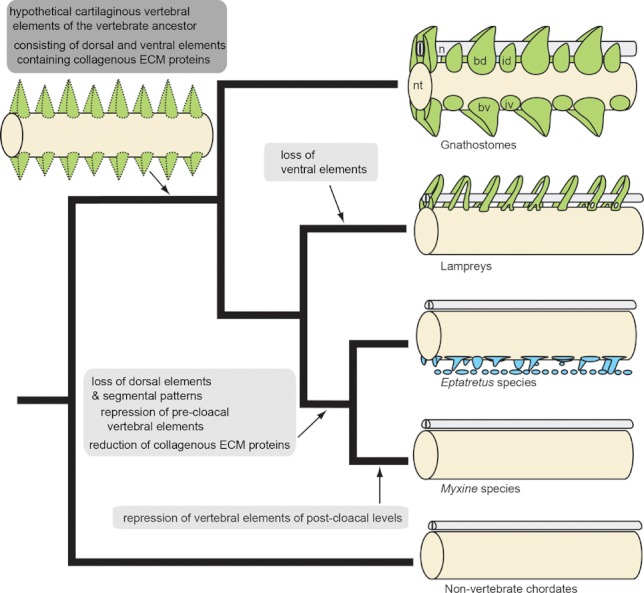
Hypothetical evolution of the vertebral elements and their extracellular matrix (ECM). This phylogenetic tree is based on molecular data. The clade of the hagfishes (including *Eptatretus* and *Myxine* species) is clustered with the lampreys as members of the monophyletic cyclostomes. The common ancestral vertebral elements of all vertebrate species are the dorsal and ventral elements, expressing collagenous ECM proteins, and their developmental mechanisms are assumed to have evolved before the divergence of the vertebrates. The collagen-rich and collagen-poor vertebral elements are in green and blue, respectively. The common ancestral vertebral elements are indicated with dotted lines. Abbreviations: bd, basidorsal; bv, basiventral; id, interdorsal; iv, interventral; n, notochord; nt, neural tube.

Except in the early phase of development, the secreted ECM proteins in the vertebral tissues differ between the hagfishes and the other vertebrates. As mentioned above, strong expression patterns of *Col2A1* are observed in the cartilaginous tissues of the lampreys and gnathostomes (Zhang et al., 2006; Ota and Kuratani, 2009), but not of the hagfishes (Ota and Kuratani, 2010). Given the long period since the divergence of the hagfishes, lampreys, and gnathostomes, the difference of the expression patterns of the *Col2A1* between the hagfishes and other vertebrates can be explained by the following hypotheses. First, Col2A1 was probably a major ECM component in the common ancestor of the vertebrates. Second, this common ancestral state has likely remained unchanged in the gnathostomes and lampreys, but *Col2A1* expression has decreased in the hagfish lineage. Finally, in the hagfish, the major ECM component probably changed from Col2A1 to noncollagenous ECM proteins, such as BGN/DCN (Fig. 6). To test these hypotheses, more detailed comparative analyses of the expression patterns of the ECM proteins and chondrogenesis-related transcription factors in the hagfish and other vertebrates are required, while simultaneously considering the evolution of the gene regulatory networks (Meulemans and Bronner-Fraser, 2007; Rychel and Swalla, 2007).

We also hypothesize that the common ancestor of the vertebrates was equipped with a complete set of two dorsal and two ventral vertebral elements, but that these degenerated secondarily in the lineage of the hagfishes and lampreys (Ota et al., 2011). This scenario is consistent with the fossil record, in that a 380-million-year-old fossil agnathan, *Euphanerops longaevus*, had vertebral elements on both the dorsal and ventral aspects of the notochord, although its phylogenetic position is still contentious (Janvier and Arsenault, 2007; Janvier, 2011). Furthermore, the cartilaginous nodule, whose morphology is reminiscent of that of the dorsal vertebral elements of the gnathostomes, is located at the most caudal level of the hagfish (Ayers and Jackson, 1900; Cole, 1905; Ota et al., 2011) (Fig. 1). Little is known about the molecular and cellular mechanisms underlying the degeneration of the dorsal vertebral element in the hagfish mid-trunk region. An expression analysis of *Msx*, which is expressed in the dorsally migrated sclerotomes in the gnathostomes, is required to resolve this problem (Monsoro-Burq et al., 1994; Christ et al., 2000).

Our study and the original description of skeletal elements by Ayers and Jackson (1900) indicate that *Eptatretus* has vertebral elements. However, this finding simultaneously raises the question of why no hagfish vertebral elements have been found in *Myxine glutinosa* (Müller, 1834; Robson et al., 2000). In fact, in the illustration from Cole shown in Figure 1A (Cole, 1905), a single isolated cartilaginous nodule is depicted on the anterior aspect of the ventral median plate of *M. glutinosa*, but no multiple nodules can be seen. This exceptional morphology of the caudal skeleton of *M. glutinosa* can be explained by repression of the development of segregated cartilaginous nodules in the postcloacal regions (Figs. 1 and 6). We propose that the expression pattern of the late *Hox* genes was evolutionarily modified in the hagfish lineage, causing the degeneration of the precloacal vertebral elements in *Eptatretus* species and also of the postcloacal vertebral elements of *Myxine* species (see Coates, 1994; Coates and Cohn, 1998; Burke et al., 1995).

Our previous and present studies have shown that the absence of hagfish vertebral elements probably represents a secondarily degenerate rather than an ancestral condition (Ota et al., 2011). Other morphological characters of the hagfish—such as the absence of a closed vascular system and multiple semicircular canals—that have long been recognized as plesiomorphic in cladistic analyses (Forey and Janvier, 1993; Shu et al., 2003; Gess et al., 2006), also seem to be secondarily degenerate characters (Kuratani and Ota, 2008). We hope that these evo-devo-based interpretations of the morphological character states of the hagfish will complement cladistic approaches, providing further insight into the evolutionary processes of the early vertebrates and their morphological features.
